# Bio‐Electrocatalytic Application of Microorganisms for Carbon Dioxide Reduction to Methane

**DOI:** 10.1002/cssc.201600963

**Published:** 2016-10-28

**Authors:** Stefanie Schlager, Marianne Haberbauer, Anita Fuchsbauer, Christine Hemmelmair, Liviu Mihai Dumitru, Gabriele Hinterberger, Helmut Neugebauer, Niyazi Serdar Sariciftci

**Affiliations:** ^1^ Linz Institute for Organic Solar CellsJohannes Kepler University LinzAltenbergerstraße 694040LinzAustria; ^2^ acib GmbHLinzAustria; ^3^ PROFACTOR GmbHSteyrAustria

**Keywords:** biosynthesis, electrochemistry, enzyme catalysis, fuel cells, reduction

## Abstract

We present a study on a microbial electrolysis cell with methanogenic microorganisms adapted to reduce CO_2_ to CH_4_ with the direct injection of electrons and without the artificial addition of H_2_ or an additional carbon source except gaseous CO_2_. This is a new approach in comparison to previous work in which both bicarbonate and gaseous CO_2_ served as the carbon source. The methanogens used are known to perform well in anaerobic reactors and metabolize H_2_ and CO_2_ to CH_4_ and water. This study shows the biofilm formation of those microorganisms on a carbon felt electrode and the long‐term performance for CO_2_ reduction to CH_4_ using direct electrochemical reduction. CO_2_ reduction is performed simply by electron uptake with gaseous CO_2_ as the sole carbon source in a defined medium. This “electrometabolism” in such microbial electrolysis cells depends strongly on the potential applied as well as on the environmental conditions. We investigated the performance using different adaption mechanisms and a constant potential of −700 mV vs. Ag/AgCl for CH_4_ generation at 30–35 °C. The experiments were performed by using two‐compartment electrochemical cells. Production rates with Faradaic efficiencies of around 22 % were observed.

## Introduction

CO_2_ reduction has gained high interest in the last decade because of research in the field of carbon capture and utilization (CCU), which is a viable strategy for cyclic carbon use. From a chemical point of view, CO_2_ is a valuable feedstock for many reactions and products such as acids, alcohols, and gases, that is, acetic acid, methanol, and methane. CO_2_ is a very stable molecule, therefore, its reduction reactions require a high energy input because of overpotentials and multielectron reduction steps.

According to the standard redox potentials shown in Equations (1)–6, the reduction of CO_2_ can be tuned toward different products. Indeed, the given potentials are much more negative in reality because of overpotentials. To lower these energy barriers, several electrochemical and biological systems have been investigated to catalyze the reduction of CO_2_. Particularly, the biological pathway of CO_2_ reduction, which uses microorganisms or enzymes, offers a biocompatible and sustainable energy storage approach. This is particularly attractive for industry because of the use of mild reaction conditions such as ambient temperature and pressure. Furthermore, biocatalysts are capable of self‐regeneration and are, therefore, highly suitable for long‐term performance systems without the loss of catalyst.^1^, ^2^, ^3^, ^4^

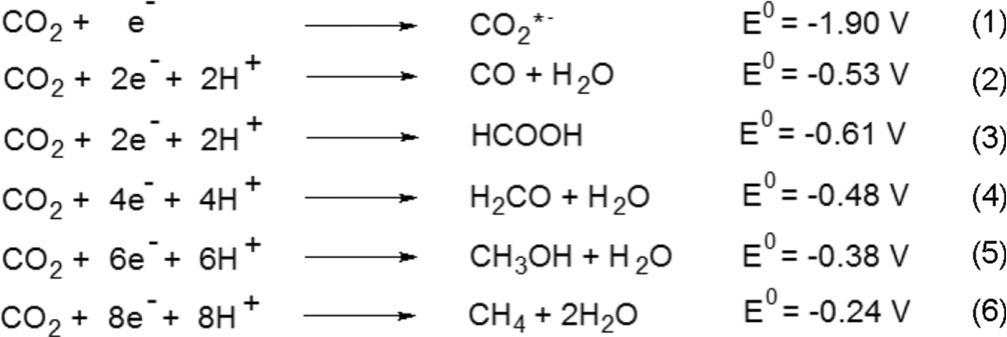



In particular, reduction reactions toward alcohols, aldehydes, and other hydrocarbons are of high interest as most of those substances can be applied directly as fuels. If we consider biocatalysts for the reduction of CO_2_, dehydrogenase enzymes are prime candidates. In 1976, Ruschig et al. reported the application of formate dehydrogenase for the reduction of CO_2_ to formate with the aid of the coenzyme NADH.^5^ Further investigations on the carboxylation of CO_2_ using dehydrogenase enzymes have been presented by Aresta and Dibenedetto.^6^ Srikanth and co‐workers used a bio‐electrochemical system (BES) with formate dehydrogenase and NADH as a cofactor for the reduction of CO_2_ to formate and further presented a detailed study on the electrochemical application of enzymes for the generation of, for example, fuels and chemicals.^7^, ^8^ Reda et al. showed a bio‐electrocatalytic approach with the electrochemical reduction of CO_2_ to formate assisted by a formate dehydrogenase (F_ate_DH) enzyme without a sacrificial coenzyme.^9^ Recently, our group has shown that electrodes with such enzymes immobilized onto it can be used efficiently for the electrocatalytic generation of higher alcohols such as butanol and the reduction of CO_2_ to methanol.^10^, ^11^ These nonliving biocatalysts, however, are each single molecules that are isolated from corresponding microorganism strains.

In a different approach, the direct application of the living biocatalysts or microorganisms, respectively, was investigated. In particular, living microorganisms have gained interest in comparison to enzymes as their products are tunable by the choice of microorganism strains and environmental parameters. Desired products can be generated with high yield and selectivity after the adaptation is complete.

Several studies have been presented for the application of microorganisms in the field of CO_2_ conversion, biosynthesis, and the production of biofuels with H_2_ equivalents added artificially. In the 1980s, Kerby and Zeikus, and Sharak Genthner and Bryant presented the growth of different microorganisms utilizing CO_2_ as the carbon source among others.^12^, ^13^ Tanner et al. showed the generation of acetate by growing *Clostridium lungdahlii* with, for example, CO, H_2_, or CO_2_.^14^ Sakai et al. presented work on ethanol and acetate generation from *Moorella sp*. and further investigated the influence of the pH and the activities of the corresponding dehydrogenase enzymes.^15^, ^16^ Liou et al. presented work on *Clostridium* strains for the generation of, for example, acetate, ethanol, and higher alcohols such as butanol from CO, CO_2_, and others, such as sugars, as the growth support and carbon source.^17^ Logan further discussed the application of microorganisms in an electrochemical system to establish microbial fuel cells.^18^ Logan and co‐workers screened studies with a focus on microbial electrosynthesis.^19^ Additionally, Kundiyana et al. investigated *Clostridium ragsdalei* for ethanol production and the influence of different parameters such as pH and temperature on their performance during fermentation.^20^ Tracy et al. depicted detailed pathways with all of the corresponding enzymatic steps in reactions of Clostridia. They showed the large variety of possible chemical products and biofuels that can be obtained by using these biocatalysts starting from CO_2_.^21^

Indeed all of these studies showed the properties and potential to generate valuable carbon‐based products using microorganisms. However, most of the approaches that aimed towards CO_2_ conversion required fermentation processes and basically focused on the metabolism of the microorganisms to generate fuels and chemicals. In our work we wanted to investigate the possibility of direct electrochemical reduction of CO_2_ using such living microorganisms grown on an electrode as biocatalyst. This offers the possibility to tune the metabolisms of the microorganisms toward a certain product, according to the potential applied, and therefore, to increase the selectivity. Furthermore, this approach depicts a method that opens ways for renewable energy storage as solar or wind energy could serve as electrical sources. Therefore, the approach presented here introduces direct electron injection into microorganisms and a charge transfer mechanism for CO_2_ reduction.

In contrast to molecular catalysts, for example, metal– organic compounds or enzymes, in which charge transfer occurs through conjugated bonds and metal ions, charge transfer in living systems, such as microorganisms, may occur outside of the cell of the living biological systems through the outer membrane. For microorganisms, it is proposed that bio‐electrochemical reactions occur mainly because of so‐called extracellular electron transfers with or without the aid of an electron shuttle: Rosenbaum et al.^22^ suggested three different cathodic extracellular electron transfer mechanisms for biocathodic microorganisms. In addition to a direct electron transfer that involves c‐type cytochrome electron transfer chains, they propose a mediated electron transfer to a periplasmic hydrogenase or a direct electron transfer that involves cytochrome–hydrogenase partnerships. Furthermore, Villano et al. discussed the influence of abiotic hydrogen generation on indirect extracellular electron transfer, which is also considered as a possible pathway for microbial cathodic reactions.^23^ Ajo‐Franklin et al. investigated charge transfer between living and nonliving organisms and tried to apply nanostructures for improved charge transport through cell membranes.^24^, ^25^ Bio‐electrocatalytic species such as microorganisms, which are capable of direct charge transfer, therefore, have gained interest for applications in electrochemical CO_2_ reduction. This work focuses on the conversion of CO_2_ to CH_4_ using hydrogenotrophic methanogens in a microbial electrolysis cell. The proposed mechanism for methanogenic mixed cultures is correlated to the well‐known mechanisms of the conversion of CO_2_ and H_2_ to CH_4_ and water in anaerobic digesters. Deppenmeier et al., Shima et al., and Ferry have discussed not only the detailed enzymological pathways, which include oxidation and reduction reactions for electron transfer within the metabolism, but also the role of metabolic groups for CH_4_ production from biomass with such methanogenic mixed cultures.^26^, ^27^, ^28^, ^29^ The metabolic pathways of these methanogens for the conversion of CO_2_ to CH_4_ can be summarized in the following overall reaction equation [Eq. (1)].

(1) $$\mathrm{CO}{}_{2}+4\;H{}_{2}\to \mathrm{CH}{}_{4}+2\;H{}_{2}O$$

However, H_2_ added artificially, which is generated before CH_4_ synthesis, makes these processes unfavorable because of the high cost and energy loss from the storage and transport of H_2_. As a different approach, the direct electrochemical reduction of CO_2_ using microorganisms as biocatalysts without the need for H_2_ added artificially is desired. This would offer direct CO_2_ reduction on a biocathode without the need for any mediator or supplementary process such as water splitting for H_2_ generation and enable the storage of renewable energies in the form of fuels and chemicals. The first study on CH_4_ synthesized bio‐electrochemically from CO_2_ without any electron shuttle or mediators was reported by Cheng et al. in 2009.^30^ Van Eerten‐Jansen et al.^31^ studied the performance of a CH_4_‐producing microbial electrolysis cell (MEC) for 188 days. The maximum energy efficiency obtained in this study was 51.3 % in a yield test. Villano et al. presented high CH_4_ production rates by using a microbial biocathode based on a hydrogenophilic methanogenic culture.^23^ In addition, they showed the possibility to establish biofilm reactors and the use of a CH_4_‐producing MEC for wastewater treatment. A bioanode able to oxidize acetate and a CH_4_‐producing biocathode were used in these studies. High acetate removal from the influent and efficient conversion to CH_4_ was shown, and 75 % of the energy was captured in the resulting CH_4_ gas.^32^, ^33^ Moreover, Sato and co‐workers discussed the possible implementation of the bio‐electrochemical conversion of CO_2_ to CH_4_ for geological storage reservoirs. Electromethanogenic CO_2_ reduction can be achieved by using biocathodes based on subsurface methanogens.^34^, ^35^, ^36^, ^37^ Furthermore, Li et al. presented the utilization of electromicrobial systems for CO_2_ reduction and showed the conversion of CO_2_ to higher alcohols by using genetically modified *Ralstonia eutropha H16*.^38^ Recently, Jiang et al. showed a bio‐electrochemical approach that used a methanogenic mixed culture for the simultaneous production of CH_4_ and CH_3_COOH from CO_2_. The CO_3_^2−^‐rich medium and gaseous CO_2_ acted as carbon‐based nutrients. They adapted microorganisms to a carbon‐nutrient‐only metabolism by reducing the amount of H_2_ added stepwise in four cycles of 10 days each.^39^ A more recent study on microbial electrosynthesis has been presented by Bajracharya et al. who used pure and mixed cultures for CO_2_ reduction. In their investigations, they applied an assembly of graphite felt and stainless steel as the cathode for the generation of acetate and CH_4_ from the conversion of bicarbonate.^40^ However, all of those approaches were performed using carbonate salts dissolved in the medium as the carbon source in place of or in addition to gaseous CO_2_. In contrast, Bajracharya et al. also presented a study on a gas diffusion biocathode to provide CO_2_ directly.^41^

In this work we were interested in using CO_2_ in its gaseous form for reduction to CH_4_. To get an idea of the bio‐electrocatalytic process, we also investigated the system in a state without any CO_2_ or bicarbonate but under inert conditions. Here we show a similar approach to the work of Jiang et al.^39^ on direct electron injection into methanogenic mixed cultures and the reduction of CO_2_ to CH_4_ [Eq. (2)].

(2) $$\mathrm{CO}{}_{2}+8\;e{}^{-}+8\;H{}^{+}\to \mathrm{CH}{}_{4}+2\;H{}_{2}O$$

However, in contrast to previous studies, we did not add CO_3_^2−^ and used gaseous CO_2_ only as the carbon source. This was done to preserve the possibility for investigations without any CO_2_ in the system but under N_2_‐saturated and, therefore, inert conditions. Furthermore, this provides a controlled supply of the carbon source and, therefore, the exact determination of the efficiency and electrochemical characterization of the system for CO_2_ reduction. These investigations proved that CH_4_ was only generated if CO_2_ was added. The approach presented here shows the conversion of CO_2_, added by purging the gas directly through the system, to CH_4_ without any other additives required. This is favorable as H_2_, an explosive gas obtained from energy‐costly processes such as the steam reforming of fossil fuels or water electrolysis, can be avoided.

## Results and Discussion

**Figure 1:**
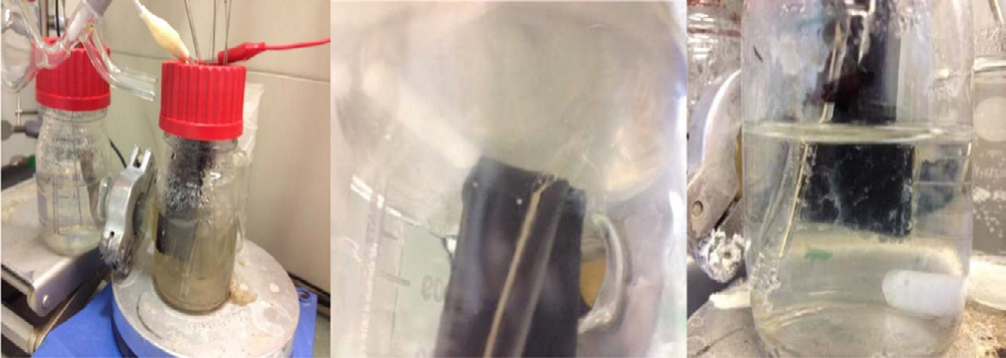
a) Cloudy medium after inoculation of microorganism suspension, b) biofilm formation after 24 h and clarified medium, c) and biofilm after 1 week (multiplying microorganisms) when the medium was exchanged.

After the inoculation of the microorganism suspension (Figure 1 a), a constant potential of −700 mV vs. Ag/AgCl was applied, and the cathode compartment was purged for approximately 5 h per day with CO_2_ and H_2_. The negative potential applied was necessary for the growth of a biofilm as the utilized microorganisms are exoelectrogenic and can immobilize on the carbon‐based electrode because of their ability to take up electrons. However, the application of a constant negative potential is required for continuous CO_2_ reduction. The reduction potential was set at −700 mV vs. Ag/AgCl according to the theoretical reduction potential of CO_2_ to CH_4_ [Eqs. (1)–(6)]. For this, the target was to use as low an overpotential as possible for the reduction to CH_4_ and to avoid competing reduction reactions such hydrogen evolution, which occurs at even lower potentials. Biofilm formation was observed after 24 h of the application of a constant negative potential (Figure 1 b). After one week, the biofilm had multiplied distinctly, and the nourishing medium was exchanged (Figure 1 c).

**Figure 2:**
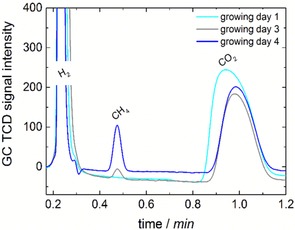
Gas chromatograms of headspace samples of the cathode compartment during the growth of the microorganisms.

During the biofilm formation, CH_4_ production was monitored. Gas chromatograms during different states of the growing process are depicted in Figure 2. The increasing CH_4_ concentration in the headspace indicates a continuous and advanced production of CH_4_.

To characterize the biocathode, cyclic voltammetry was performed before and after the adaption was completed to investigate the redox processes associated with the microorganisms.

**Figure 3:**
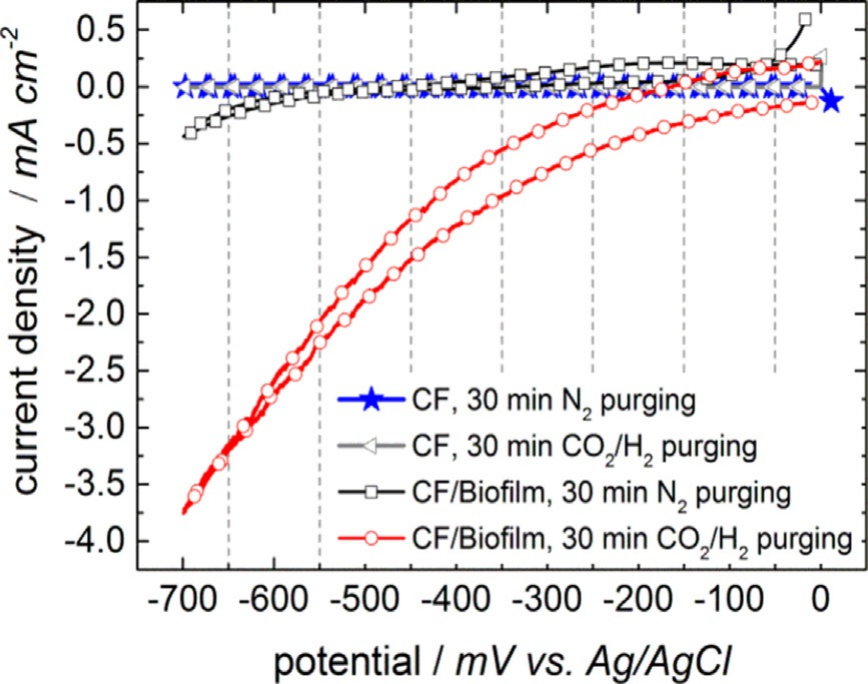
CVs of the biocathode in the nonadapted state. The biocathode (CF/biofilm) was characterized electrochemically with a scan rate of 1 mV s^−1^ after purging and saturating the system with N_2_ or CO_2_/H_2_ respectively. For comparison, the same measurements were performed for a pristine carbon felt electrode without biofilm (CF).

In the abiotic (noninoculated) MEC with a pristine carbon felt electrode, redox peaks were not detected either with N_2_ or with CO_2_/H_2_ purging (Figure 3, gray line with triangles and blue line with stars). In the biotic (inoculated) nonadapted MEC a distinct increase in the reductive current from an offset of −200 mV vs. Ag/AgCl and a peak value at −700 mV vs. Ag/AgCl was observed. This reductive peak correlates with the reduction of CO_2_ to CH_4_ at a theoretical potential of −0.446 V vs. Ag/AgCl (−0.24 V vs. normal hydrogen electrode (NHE); Figure 3, dotted red line) with a low overpotential of only 0.25 mV compared to the theoretical potential. For the N_2_‐purged system a small increase was observed as well (Figure 3, black line with squares), which is assumed to be because of insufficient flushing with N_2_ before the measurement and the removal of CO_2_ in the cathode compartment, respectively. However, the reduction current density is much higher in the case of CO_2_/H_2_ purging. This behavior supports the expectation of electrochemically active microorganisms established on the carbon felt electrode.

**Figure 4:**
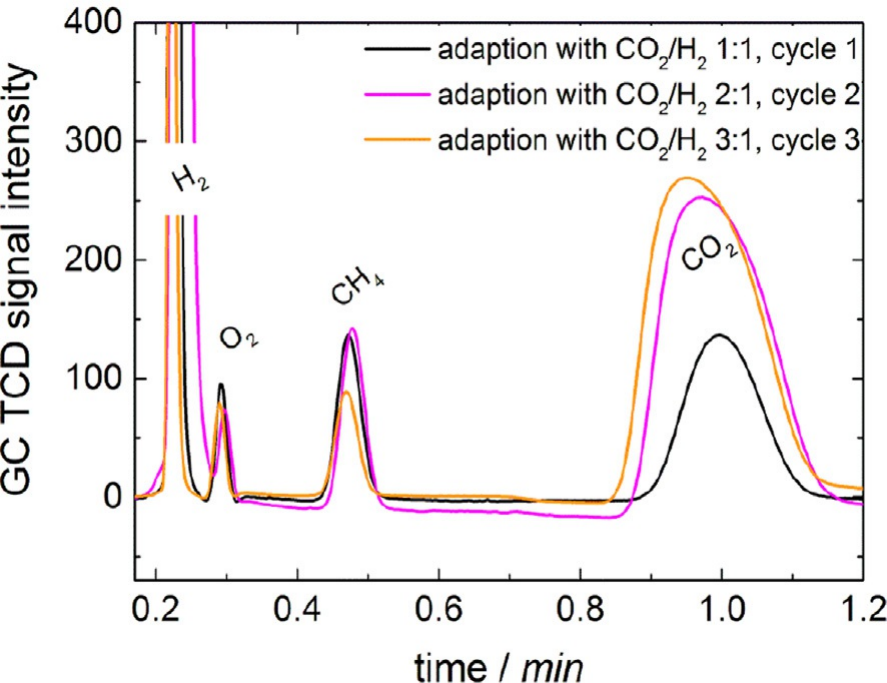
Gas chromatograms of the development of the headspace gas constitution during adaption with CO_2_ and H_2_. The concentration of H_2_ added was reduced with every cycle. Adaption was performed within three cycles over 3 weeks.

The first adaption process was performed using the technique of the successive decrease of the amount of H_2_ purged through the system in three cycles. Each cycle consisted of 5 days of purging with a certain ratio of CO_2_/H_2_ and 2 days of no purging. During the adaption process, the CH_4_ production of the microorganisms on the cathode was investigated continuously by measuring headspace gas samples by using GC, and the chromatograms of each cycle are shown in Figure 4.

Even though the amount of H_2_ added was reduced continuously, CH_4_ generation was not affected distinctly, as can be seen from the rather uniform peak and, therefore, comparable concentration of CH_4_ during all three cycles.

**Figure 5:**
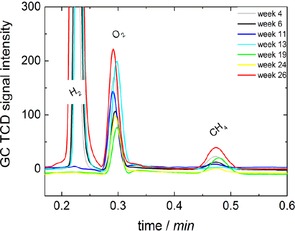
Gas chromatograms that show long‐term performance after completed adaption with CO_2_ and H_2_ (3 weeks). In general, the CH_4_ production decreased compared to the performance immediately after adaption. However, CH_4_ generation was rather constant over 22 weeks of performance despite its low concentration, as seen for the CH_4_ peaks at 0.48 min by using GC.

The long‐term performance of the microbial electrosynthesis of CH_4_ was investigated for the adapted MEC. The only carbon source to be reduced to CH_4_ was gaseous CO_2_ bubbled through the cathode compartment regularly. The CH_4_ production from long‐term microbial electrolysis over 22 weeks is shown in Figure 5. The CH_4_ concentration in the headspace was rather constant.

In addition to CH_4_, H_2_ generation was observed, which is expected to be produced by the microorganisms themselves. As there was a steady potential applied to the system, we would expect water splitting—independent of the microorganisms on the electrode—with a constant generation and constant amount of H_2_. However, H_2_ was not generated with a pristine carbon felt electrode at the same potential, and it is clear from the results presented in Figure 5 steady H_2_ evolution was not observed. We propose that the CO_2_ reduction to CH_4_ using the bioelectrode occurs either through a direct electrochemical reduction because of electron uptake or through indirect electrochemical reduction because of intermediate H_2_ generation by the microorganisms and subsequent conversion with CO_2_ to CH_4_.

Although the performance of the microbial electrolysis cell was rather constant over several weeks according to CH_4_ generation from CO_2_ reduction, efficiencies were low. Therefore, we tried improve the process. For this a second adaption, a different approach using glucose and an enhancement of the biofilm on the carbon felt cathode was examined.

To improve biofilm formation, more microorganism suspension was added to the cathode compartment.

**Figure 6:**
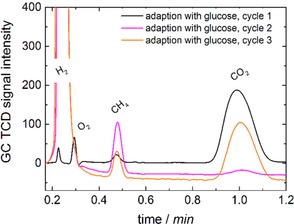
Gas chromatograms that show the development of CH_4_ generation with the adaption of the microorganisms using glucose.

For the second adaption process, glucose (0.1 mL saturated aqueous solution) was added to the cathode compartment instead of reducing the H_2_ purged through the cathode compartment continuously (Figure 4). Additionally, the cathode compartment was purged regularly with CO_2_. The adaption process using glucose within three cycles was monitored (Figure 6). As glucose and CO_2_ served as carbon‐based nutrients but the amount of glucose in the electrolyte medium was depleted during the three cycles, gaseous CO_2_ was the remaining and only carbon source after adaption and, therefore, the source for the reduction to CH_4_. In contrast to adaption with H_2_, the CH_4_ generation first increased and then stabilized at a slightly lower amount. The addition of glucose provides a simple way for adaption without the need for H_2_ addition. This is favorable as H_2_ can be avoided.

**Figure 7:**
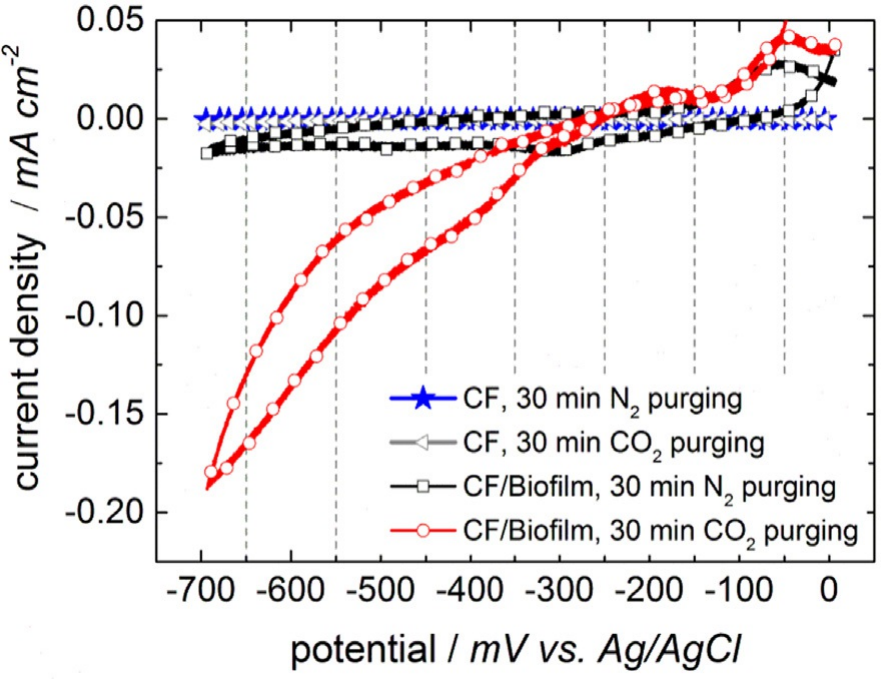
CVs of the adapted biocathode (CF/biofilm) in N_2_‐ and CO_2_‐saturated systems recorded at a scan rate of 1 mV s^−1^. An increase in the reductive current is only observed after the system was purged with CO_2_. Further comparison with a pristine carbon felt electrode without any biofilm (CF) supports that CO_2_ reduction only occurs if microorganisms are present.

Cyclic voltammograms (CVs) were recorded for the characterization of the final biocathode after the completion of the second adaption with glucose (Figure 7). There is an increase in the reductive current from −300 mV vs. Ag/AgCl observed for the CO_2_‐saturated system (dotted red line). Saturation with N_2_ and experiments without biofilm did not deliver an increase in reductive current, which indicates that the predominant reaction of the microbial electrolysis is the reduction of CO_2_ to CH_4_. In comparison to values obtained for CVs recorded for the nonadapted state (Figure 3), current densities are lower by approximately an order of magnitude. However, the CVs displayed in Figure 7 cannot be compared directly as not only the CV conditions were changed (only CO_2_ purging instead of CO_2_/H_2_) but also the constitution of the mixed culture and the metabolism of the microorganisms was modified after reinoculation, second adaption, and further weeks of long‐term performance. Nevertheless, the electrochemical characterization of the biocathode showed that reduction reactions that take place are only observed if CO_2_ and microorganisms were both present.

**Figure 8:**
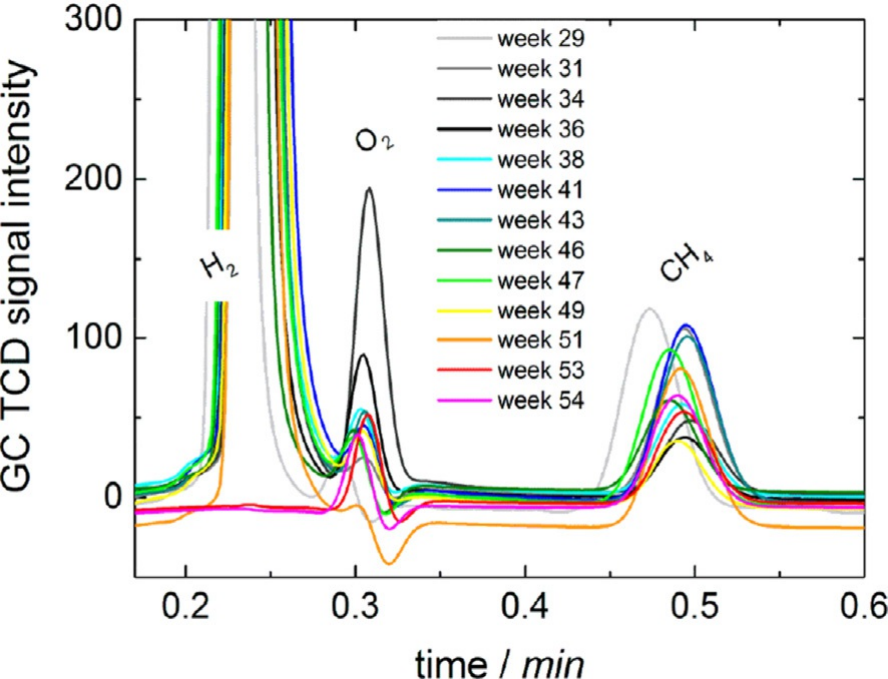
Gas chromatography records for the long‐term performance of MEC1 after repeated adaption with glucose. The production of CH_4_ from CO_2_ reduction shows a remarkable increase in comparison to results from long‐term performance before adaption with glucose.

The long‐term performance of the final adapted biocathode was monitored by investigating the constitution of the headspace gas samples over several weeks. The gas chromatograms measured over 25 weeks after the adaption with glucose are presented in Figure 8.

As observed previously after adaption with CO_2_/H_2_, the CH_4_ generation was rather constant during long‐term performance after the glucose adaption of the biocathode. For H_2_ generation, the same effects in terms of nonconstant concentrations were observed as for the first long‐term investigation (Figure 5). It is expected that H_2_ is not only produced by the microorganism but also partly used by the microorganism for its metabolism and the reduction of CO_2_ to CH_4_. The direct electrochemical reduction of CO_2_ and the indirect electrochemical reduction of CO_2_ using intermediate hydrogen is, therefore, not distinguishable.

**Figure 9:**
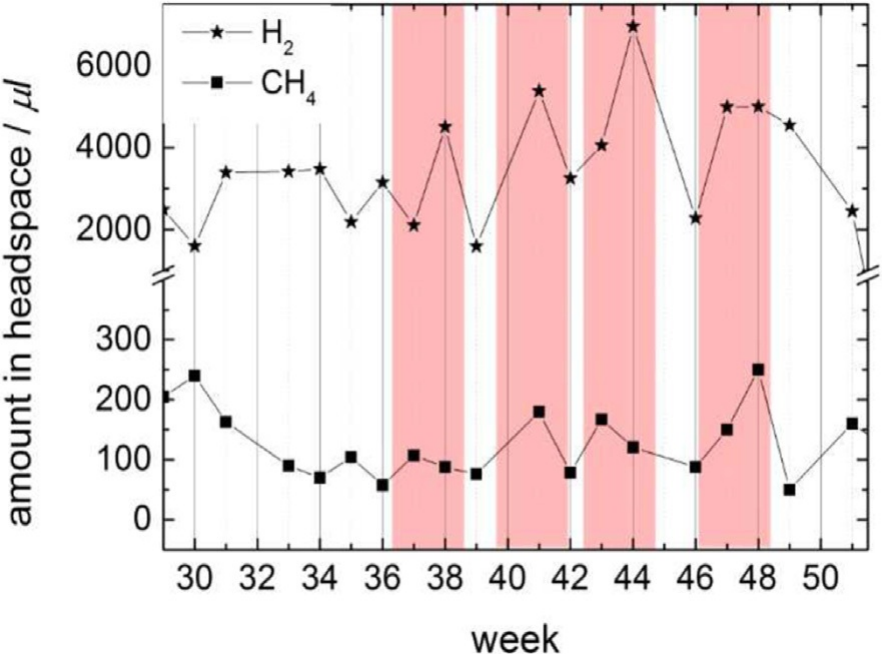
Correlation of the amounts of H_2_ and CH_4_ in the headspace of the cathode compartment during long‐term performance after the second adaption.

The correlation of the detected amounts of H_2_ and CH_4_ from headspace gas samples of the cathode compartment of the MEC over that time shows that fluctuations of H_2_ and CH_4_ concentration were mainly parallel (Figure 9). The peak values of concentrations for both CH_4_ and H_2_ were reached at approximately the same time during the long‐term performance.

Potentiostatic electrolysis for 4 h at a potential of −700 mV vs. Ag/AgCl after biofilm improvement and completed adaption delivered an overall Faradaic efficiency of around 22 %, calculated according to the charge consumed (39 C) and the amount of CH_4_ and H_2_ generated (17.5 μL of CH_4_ and 930 μL of H_2_), which was detected by using GC in the headspace volume of the cathode compartment.

These results show an efficient, biological approach of CO_2_ reduction to a valuable fuel at rather high efficiencies and with gaseous CO_2_ as the only carbon source. Microbial electrosynthesis has been shown to be applicable in a long‐term and continuous run with a rather constant production rate of CH_4_. Furthermore, the efficiency can be tuned by enhancing the biofilm formation and increasing the amount of microorganisms immobilized on the cathode. It is also expected that mixed cultures could be modified with regard to their constitution and, therefore, their ability to metabolize CO_2_ sufficiently to CH_4_ by tuning the adaption method for a CO_2_‐only process. These observations depict a very interesting approach for CO_2_ recycling. Additionally, such electrochemical processes could be driven by renewable energy sources and, therefore, represent an attractive, sustainable method for renewable energy storage.

## Conclusions

We showed the utilization of methanogenic microorganisms, from digestate, for the reduction of CO_2_ to CH_4_ in a microbial electrocatalytic synthesis approach. Microbial electrolysis cells offer great potential for sustainable, highly efficient, and selective CO_2_ reduction. CO_2_ was reduced in a process in which microorganisms were grown on a carbon‐based cathode for heterogeneous electrocatalysis. CH_4_ was generated by direct electron injection into biocatalysts through a direct or indirect electrochemical reduction pathway with Faradaic efficiencies of approximately 22 %. For this neither H_2_, carbonate, nor any other carbon source except gaseous CO_2_ was added artificially. In addition, no mediator or supplementary cofactors were required. This is new and different to previous studies, for example, that of Jiang et al., in which bicarbonate was used as a carbon source in the electrolyte solution.^39^ Therefore, they could not fully comprehend if the CH_4_ generation was from bicarbonate or gaseous CO_2_ flushed through the system or investigate the properties in a system without the addition of any carbon source. Moreover, we showed an extraordinary long‐term performance of one year of continuous CH_4_ and H_2_ generation, which even extended earlier long‐term investigations of, for example, van Eerten‐Jansen et al.^31^ If H_2_ was produced during these electrochemical processes, it could act as a supply for the metabolism of the microorganisms. However, the direct electrochemical reduction of CO_2_ and the indirect electrochemical reduction of CO_2_ using intermediate H_2_ are not distinguishable.

The application of living organisms is advantageous because of their self‐regeneration and adaptability to certain conditions. Here, we present a simple and efficient process that is suitable for a long‐term performance of several months with continuous and stable production rates.

Additionally, an improvement of the biocathode was obtained and a second more favorable adaption technique for a CO_2_‐only process was investigated. Here, for the first time, two different adaption techniques were applied during a continuously running experiment.^12^, ^30^ We found a convenient improvement of an existing technology that will gain interest in the topics of renewable and sustainable energy, CO_2_ reduction, and energy storage with biocatalysts.

## Experimental Section

### Setup

All experiments were performed by using a two‐compartment cell with separate anode and cathode compartments to avoid the diffusion of oxygen generated anodically and the reoxidation of products generated cathodically. Both compartments were sealed with silicon septa to enable gas purging, sample withdrawal, and to guarantee anaerobic conditions for the microorganisms. Carbon felt (CF) with a size of 2.5×6×0.6 cm (the active area dipped in the electrolyte solution was approximately one third of the electrode, and the Pt wire was not immersed in the electrolyte solution), with a Pt wire as the electrical contact served as working electrode. Pt foil was applied as the counter electrode. The potentials were applied versus a Ag/AgCl reference electrode mounted in the cathode compartment. The anode compartment contained phosphate buffer of pH 7 as the electrolyte solution and was purged with N_2_ regularly to prevent oxygen diffusion. For the cathode compartment, a temperature of 30–35 °C was chosen, and a nutrient medium that consisted of phosphate buffer, vitamins, and trace elements with pH 7 was used as the electrolyte solution. The medium contained the following ingredients (per liter): KH_2_PO_4_ (3 g), K_2_HPO_4_⋅H_2_O (2.5 g), NH_4_Cl (310 mg), NaCl (130 mg), trace element solution (12.5 mL), and vitamin solution (5 mL). The trace element solution contained: HCl (25 %; 7.7 m, 10.00 mL), FeCl_2_⋅4 H_2_O (1.50 g), ZnCl_2_ (70.00 mg), MnCl_2_⋅4 H_2_O (100.00 mg), H_3_BO_3_ (6.00 mg), CoCl_2_⋅6 H_2_O (190.00 mg), CuCl_2_⋅2 H_2_O (2.00 mg), NiCl_2_⋅6 H_2_O (24.00 mg), Na_2_MoO_4_⋅2 H_2_O (36.00 mg), and distilled water (990.00 mL). The vitamin solutions consisted of the following (per liter): biotin (2.00 mg), folic acid (2.00 mg), pyridoxine hydrochloride (10.00 mg), thiamine hydrochloride dihydrate (5.00 mg), riboflavin (5.00 mg), nicotinic acid (5.00 mg), d‐calcium pantothenate (5.00 mg), Vitamin B12 (0.10 mg), *p*‐aminobenzoic acid (5.00 mg), and lipoic acid (5.00 mg).

### Enrichment of microorganisms

The source for the microbial inoculum was digestate collected from a wastewater treatment plant Asten (Austria). The digestate was centrifuged at 4000 rpm for 10 min. The supernatant was cultivated in headspace vials in a nutrient medium, prepared as described above, in a H_2_/CO_2_ atmosphere (4:1) at 37 °C under orbital shaking.

### Biofilm formation on the cathode

For the inoculation of the microorganisms, the system was purged with N_2_ gas to achieve appropriate anaerobic conditions for the methanogenic mixed culture. The enriched microorganism suspension (9 mL, 10 vol %) was inoculated into the cell. The cathode chamber was purged with CO_2_ and H_2_ of a ratio of approximately 1:1, and a potential of −700 mV vs. Ag/AgCl was applied. After the clarification of the electrolyte solution caused by the attachment of the microorganisms onto the electrode as a biofilm, the medium was refreshed in the cathode compartment (Figure 1).

### Adaption of the biocathode

The inoculated microorganisms were first adapted by nourishing with CO_2_/H_2_ and reducing the H_2_ amount continuously over three cycles (1:1, 2:1, and 3:1). For every adaption step, the ratio was kept for 5 days and for 5 h every day followed by 2 days of no purging. The medium of the compartment was refreshed every 2–3 weeks to provide an appropriate nutrient solution for the microorganism. Furthermore, a potential of −700 mV vs. Ag/AgCl was applied constantly. Product generation was investigated daily by measuring the gas composition of the headspace by injecting 2 mL of headspace gas into a gas chromatograph (Thermo Scientific, Trace GC Ultra) with a gas‐tight syringe. After the long‐term performance of 28 weeks, microorganism suspension was inoculated into the cell to enhance the biofilm on the cathode and to improve performance of the cell. Adaption to a CO_2_‐only performance (without any H_2_ purging required) was then undertaken in a different approach. For this, 0.1 mL of saturated glucose solution was added, and the cathode compartment was purged with CO_2_ for approximately 5 h per day. The amount of glucose in the system decreased automatically because of the microorganisms themselves as glucose was metabolized to CH_4_ and, moreover, by refreshing the medium in the cathode compartment after 2 weeks to provide appropriate conditions (vitamins, trace elements) for the microorganisms.

### Electrochemical characterization

The biofilm electrode was characterized electrochemically in its nonadapted state after the first inoculation, after the biocathode was improved, and after the second adaption with glucose was completed to a process with CO_2_ as the only carbon source. Electrochemical characterization was performed by using CV. CVs were recorded after purging the cell with N_2_ and CO_2_/H_2_ (1:1) or CO_2_, respectively, for comparison. Furthermore, also a blank CF electrode was characterized by using CV under the same conditions. CVs were recorded by using a Jaissle Potentiostat‐Galvanostat IMP 88 PC‐R or 1030 PC.T., and electrolysis measurements were realized by using a Jaissle Potentiostat P‐M 100. For CV, potentials were swept between 0 and −700 mV vs. Ag/AgCl with a scan rate of 1 mV s^−1^.

### Electrolysis for efficiency determination

Potentiostatic electrolysis at −700 mV vs. Ag/AgCl was conducted for 24 h after the first adaption with CO_2_ and H_2_ and for 4 h after completed glucose adaption of the microorganisms to determine Faradaic efficiencies. CO_2_ gas was the sole carbon source to be reduced sufficiently to CH_4_ for both electrolysis experiments. For potentiostatic electrolysis, the cathode chamber was purged with N_2_ for 1 h to ensure inert conditions and, subsequently, with CO_2_ for 2 h to saturate the electrolyte solution. Headspace samples were analyzed before and after electrolysis by using GC. The charge [C] consumed during potentiostatic electrolysis was calculated from the current over time curve and correlated, by means of the eight‐electron reduction of CO_2_ shown in Equations (1)–(6), to the amount of CH_4_ produced for the calculation of the Faradaic efficiency.
